# Prognostic significance of negative conversion of high-risk Human Papillomavirus DNA after treatment in Cervical Cancer patients

**DOI:** 10.7150/jca.46683

**Published:** 2020-08-10

**Authors:** Qingqing Chen, Runjun Shi, Zhengcao Liu, Zhouhong Shi, Ke Gu, Jie Chen, Yan He, Ying Li, Jinchang Wu, Shengjun Ji, Jundong Zhou, Jiahao Zhu

**Affiliations:** 1 Department of Radiotherapy & Oncology, The Affiliated Suzhou Hospital of Nanjing Medical University, Suzhou, China.; 2 Department of Radiotherapy & Oncology, Affiliated Hospital of Jiangnan University, Wuxi, China.; 3 Department of Gynecology, The Affiliated Suzhou Hospital of Nanjing Medical University, Suzhou, China.

**Keywords:** High-risk Human Papillomavirus DNA, Prognosis, Cervical Cancer

## Abstract

**Objective:** To evaluate the prognostic value of conversion of high-risk human papillomavirus (HR-HPV) status after treatment for cervical cancer.

**Methods:** A total of 112 cervical cancer patients with HR-HPV positivity without distant metastasis treated with surgery or radical concurrent radiochemotherapy were enrolled. HR-HPV status was analyzed before and after treatment and at the time point of recurrence or metastasis. Log-rank tests and Cox proportional hazard models were used to evaluate the association between conversion of HR-HPV status after treatment and survival.

**Results:** Eighty-four (75%) patients had negative conversion HR-HPV (ncHR-HPV) after treatment and twenty-eight (25%) were persistent positive HR-HPV (ppHR-HPV). The negative conversion rate was 75.8% in patients who received surgical treatment and 71.4% in patients who received radical concurrent radiochemotherapy. There was no significant difference between the two groups (χ^2^=0.000, *P*=1.000). There was no significant correlation between HR-HPV conversion after treatment with age (χ^2^=0.616, *P*=0.252), FIGO stage (χ^2^=0.051, *P*=0.823) and pathological type (χ^2^=0.000, P=1.000). Univariate analysis showed that treatment regimen and ncHR-HPV was closely related to progression-free survival (PFS) and overall survival (OS) of cervical cancer patients. Multivariate COX regression model showed that treatment regimen (HR=3.57, 95% CI: 1.57-8.11, *P*=0.002) and ncHR-HPV (HR=5.14, 95% CI: 2.32-11.46, *P<*0.001) were independent prognostic factors for PFS, while only ncHR-HPV (HR=12.56, 95% CI: 3.54-44.65, *P<*0.001) was an independent prognostic factor for OS. The presence of ppHR-HPV after treatment (χ^2^=14.827, *P*<0.001) was associated with recurrence and metastasis. Eleven of the patients with ncHR-HPV after treatment had recurrence or metastasis, and HPV reinfection was not detected in any of them.

**Conclusion:** ncHR-HPV after treatment in cervical cancer patients indicated better PFS and OS, while ppHR-HPV indicated worse prognosis and high risk of recurrence or metastasis. For patients with ncHR-HPV after treatment, continued HPV screening may not predict recurrence or metastasis. This study suggested that HR-HPV monitoring is necessary for ppHR-HPV patients after treatment but may not be for ncHR-HPV patients. However, further large and multi-center prospective studies should be performed to confirm these findings.

## Introduction

Cervical cancer is one of the most common gynecologic cancers worldwide and causes thousands of cancer-related deaths in women, and there are about 500,000 new cases of cervical cancer in the world each year 1. China accounts for about one-third of the world's total annual new cases 2. Despite advances in treatment development during the past decade, 20% to 40% of cervical cancer patients will still have local recurrence or distant metastases, especially during the initial two years after treatment 3-4. As it is well known, the International Federation of Gynecology and Obstetrics stage (FIGO stage) is a prognostic factor of cervical cancer, but significant differences in prognosis are often observed for the same stage. Therefore, it is necessary to seek additional biomarkers with significant clinical prognostic value for cervical cancer.

Infection with human papillomavirus (HPV), especially with high-risk HPV(HR-HPV), including HPV16, 18, 31, 33, 35, 39, 45, 51, 52, 56, 58, 59, 68, 73 and 82, is the most important risk factor associated with cervical cancer 5-7. It has been reported that more than 90% of patients with invasive cervical carcinoma were infected with HPV 8. In the past years, a large number of studies focused on the correlation between HPV status before treatment and prognosis in patients with cervical cancer 9-13. Though some studies have shown that cervical cancer patients with HR-HPV positive could become negative after treatment (negative conversion), few studies were concerned with HR-HPV status after treatment. Intharaburan et al. reported that HPV positive after treatment was associated with persistent and recurrent disease and this oncogenic virus may be was a biomarker for pelvic recurrence 14. However, the prognostic value of the negative conversion HR-HPV (ncHR-HPV) is yet to be reported. Therefore, we assessed the association of the ncHR-HPV with overall survival (OS) and progression free survival (PFS) in cervical cancer patients. Additionally, the association between persistent positive HR-HPV (ppHR-HPV) after treatment and recurrence or metastasis in cervical cancer was evaluated.

## Materials & Methods

### Patients

For this retrospective study, we collected data of cervical cancer patients who had been diagnosed with HR-HPV positive before treatment in The Affiliated Suzhou Hospital of Nanjing Medical University. Inclusion conditions: (1) Cervical cancer was confirmed by pathology. Patients received surgery or radiotherapy or chemotherapy according to the National Comprehensive Cancer Network (NCCN) Cervical Cancer guidelines. (2) HPV of the patients was positive and the classification was clear before treatment. (3) There is no history of HPV vaccination. (4) No distant metastasis was confirmed by imaging examination. Exclusion criteria: (1) Patients with no pathology. (2) HR-HPV was negative or unclear before treatment. (3) Patients without HPV test after treatment. (4) Patients who lost follow-up and did not follow up regularly. (5) Patients have suffered from other malignant tumors in the past. All patients were confirmed in accordance with the pathologic evidence and had the Karnofsky Performance Status (KPS) of more than 70. Staging was based on the criteria of FIGO 2009. The basic information of these patients was collected, including age, clinical stage, pathological classification, and treatment regimen. Patients were divided into two groups with ncHR-HPV and ppHR-HPV respectively depending on conversion of their HPV status before and after treatment.

All procedures performed were in accordance with the ethical standards of the responsible committee on human experimentation (institutional and national) and with the Helsinki Declaration of 1964 and later versions. This study was approved by the Institutional Review Board of The Affiliated Suzhou Hospital of Nanjing Medical University (No. KL901060). Informed consent was obtained from all individual participants included in the study or their family.

### HPV genotyping

A cervical brush was fully inserted into the cervical canal and the cervical canal was gently rotated five laps clockwise. Cervical exfoliated cells were collected inside and outside the cervix and brush was placed in the preservation solution to prepare for HPV testing. We used HPV nucleic acid amplification test kit for Cape biochemical company (Guangzhou, China). HPV DNA in cervical secretions was detected with hybridization micro array technology (Hybri Max) detection. It Can detect 21 kinds of HPV subtypes, including 15 kinds of high-risk subtype (16, 18, 31, 33, 35, 39, 45, 51, 52, 56, 58, 59, 68, 73 and 82) and 6 kinds of low-risk subtype (6, 11, 42, 43, 44, CP8304). All respondents underwent gynecological examination and were collected of cervical secretions by a professional gynecologist. With a cervical brush was fully inserted into the cervical canal, the cervical canal was gently rotated 5 laps by clockwise. Cervical exfoliated cells were collected inside and outside the cervix, and brush was placed in the preservation solution to prepare for HPV testing. Mentioned the use of genome extraction kit DNA, PCR amplification (using AB 17300-based PCR amplification), hybridization, and got HPV typing results obtained by color. HPV in cervical secretions was detected for each patient every three month after treatment and at the time of recurrence or metastasis.

If one or more HPV subtype was positive in 21 kinds of subtypes, it represented HPV DNA testing was positive. All of the negative for HPV DNA was determined to be negative of HPV DNA. If one or more high-risk HPV subtype was positive in 21 kinds of subtypes, it represented high-risk HPV DNA testing was positive.

### Treatment

Patients with early-stage cervical cancer (FIGO stage IB1-IIA) underwent radical hysterectomy and pelvic lymph node dissection. Postoperative pelvic radiotherapy ± concurrent chemotherapy was administered to patients at high risk for recurrence. Patients with locally advanced cervical cancer (FIGO stage IIB-IV) underwent concurrent radiochemotherapy (CCRT). External beam radiotherapy (EBRT) was delivered to the whole pelvis with 45-50Gy in 25 fractions for the postoperative patients. In the definitive setting, HDR ICR was delivered twice a week in 5 fractions, with a total dose of 30Gy. The prescription of dose was given to point A. The cisplatin with 75mg/m^2^ was administered for two courses during radiotherapy.

### Follow-up

Follow-up examinations were performed at regular intervals: three monthly during the first three years, six monthly during the following year. OS was defined as the time between the date of diagnosis and the date of cervical cancer-related death or the last follow-up. PFS was measured from the date of diagnosis to the date of recurrence, distant metastasis, or the last follow-up.

### Statistical methods

The χ^2^ test was performed to compare categorical variables. Associations between HR-HPV status and OS and PFS were analyzed using Kaplan-Meier curves and were compared using the log-rank test. Multivariate analysis for OS and PFS was performed using Cox proportional hazards regression models. The statistical analyses were performed with SPSS 20.0 statistical software (IBM Corporation, Armonk, NY, USA). A *P*-value < 0.05 was considered statistically significant.

## Results

### Patient characteristics

In total, 112 patients were included in the current study with their characteristics shown in **Table 1.** The average age was 53.0 ± 10.2 years, with a range of 32 to 88 years. Of these, 69 (61.6%) patients were with early tumor stages (FIGO IA-IIA2), 43 (38.4%) patients were with advanced tumor stages (FIGO IIB-IV). Furthermore, 91 (56.8%) patients received surgery and 21 (43.2%) patients received concurrent radiochemotherapy. Some of the patients with advanced tumor stages were still undergoing surgery. A total 25 patients (22.3%) had recurrence and metastases, and 9 patients (8.0%) died due to disease progression.

### Association between ncHR-HPV and clinicopathologic features

In the total patients, 84 patients had ncHR-HPV (75%) after treatment. Of the 91 patients who underwent surgery, 69 patients had ncHR-HPV (75.8%). The 21 patients who underwent radical concurrent radiochemotherapy, 15 Patients had ncHR-HPV (71.4%). There was no significant difference between the two groups (χ^2^=0.000, *P*=1.000). There was no significant correlation between HR-HPV conversion after treatment neither with age (χ^2^=0.616, *P*=0.252), FIGO stage (χ^2^=0.051, *P*=0.823), or pathological type (χ^2^=0.000, *P*=1.000) (**Table 2**).

### OS and PFS according to the HR-HPV conversion status

**Figure 1:**
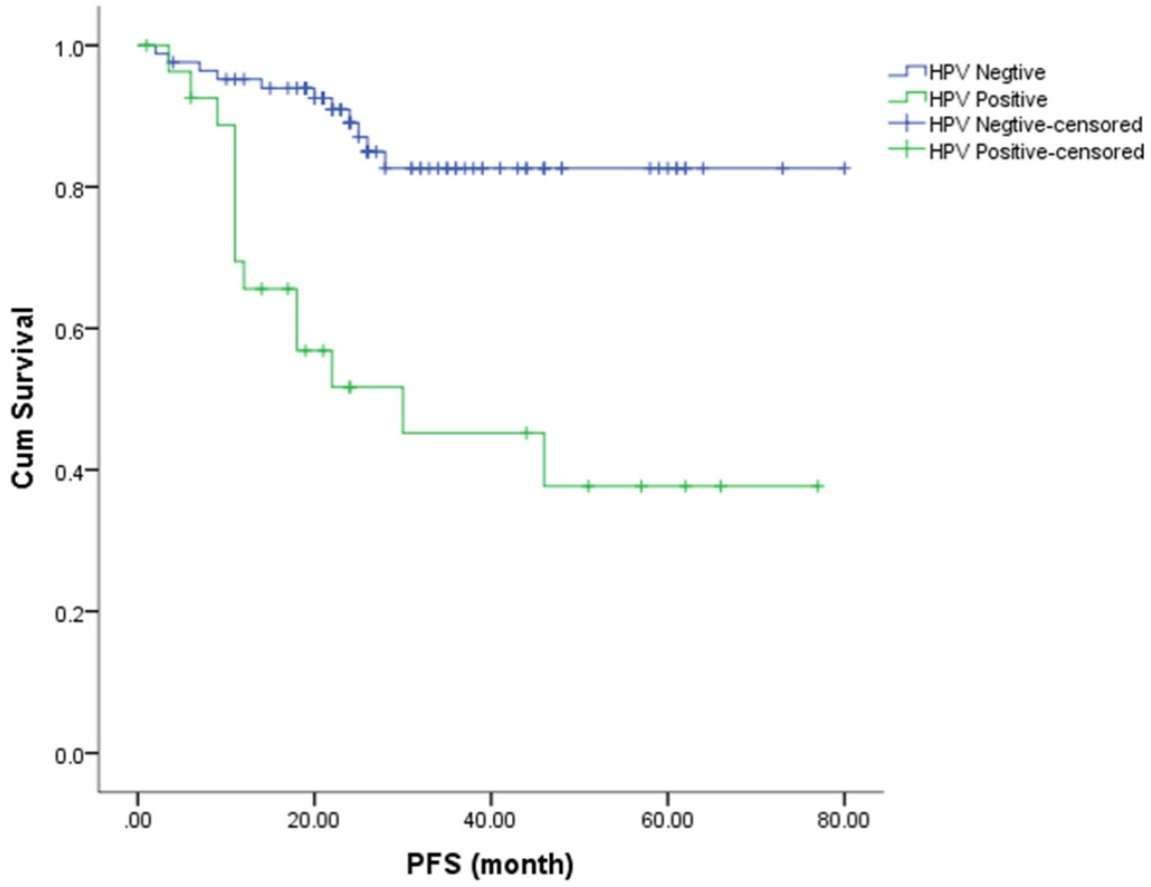
Kaplan-Meier analysis shows the PFS for patients with cervical cancer.

**Figure 2:**
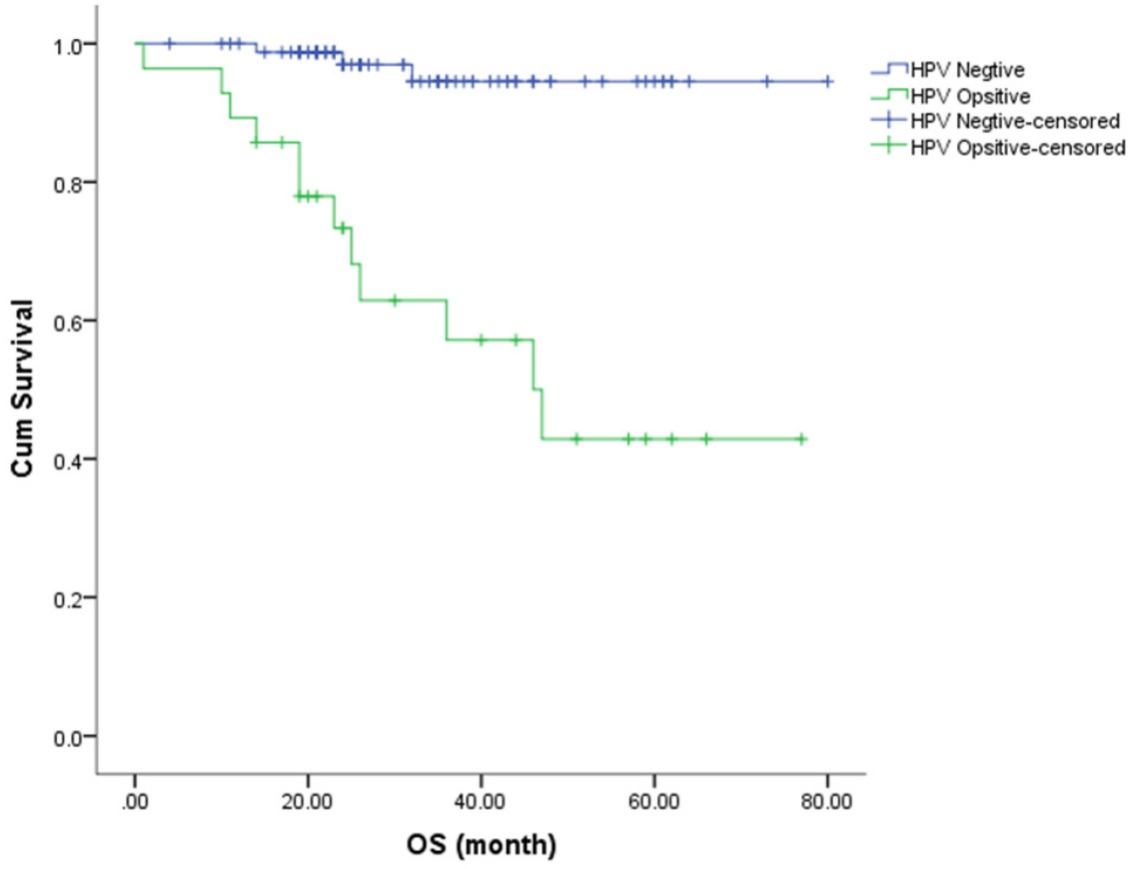
Kaplan-Meier analysis shows the OS for patients with cervical cancer.

The Kaplan-Meier method was used to estimate the OS and PFS curves, which were stratified according to the HR-HPV status after treatment (**Figures 1 & 2**). The 5-year rate for OS and PFS associated with the HR-HPV conversion status after treatment were calculated. The 5-year OS rate was 94.5% and 57.1% in the ncHR-HPV and ppHR-HPV groups, respectively (HR=22.821, 95% CI=5.142 to 101.304, *P*<0.001). The 5-year PFS rate was 82.5% and 45.2% in the ncHR-HPV and the ppHR-HPV groups, respectively (HR=4.923, 95% CI=1.866 to 12.989, *P*<0.001). In brief, the patients with ncHR-HPV had better OS and PFS than those of patients with ppHR-HPV.

### Univariate and multivariate cox regression survival analyses

Univariate analysis showed that treatment regimen (HR=0.29, 95% CI: 0.10-0.90, *P*=0.002) and ncHR-HPV status (HR=0.21, 95% CI: 0.08-0.53, *P*<0.001) were closely correlated with PFS in cervical cancer patients. Multivariate COX regression model showed that treatment regimen (HR=3.57, 95% CI: 1.57-8.11, *P*=0.002) and ncHR-HPV status (HR=5.14, 95% CI: 2.32-11.46, *P<*0.001) were independent prognostic factors for PFS (**Table 3**).

Univariate analysis showed that treatment regimen (HR=0.35, 95% CI: 0.10 -1.27, *P*=0.03) and ncHR-HPV (HR=0.08, 95% CI: 0.03-0.26, *P<*0.001) were closely correlated with OS in cervical cancer patients. Multivariate COX regression model showed that only ncHR-HPV (HR=12.56, 95% CI: 3.54-44.65, *P<*0.001) was an independent prognostic factor for OS (**Table 4**).

### HR-HPV conversion status and recurrence or metastasis

Total of 25 patients had recurrence and metastasis, and 13 of them had ppHR-HPV. The presence of ppHR-HPV after treatment (χ^2^=14.827, *P*<0.001) was associated with recurrence or metastasis (**Table 5**). Total of 12 patients with ncHR-HPV after treatment had recurrence or metastasis, and in none of them HPV reinfection was found.

## Discussion

In the present study, we evaluated the prognostic value of HR-HPV conversion after treatment for patients with cervical cancer. The results showed that ncHR-HPV after treatment was independent prognostic factor for PFS and OS. The presence of ppHR-HPV after treatment was associated with recurrence and metastasis. Eleven of the patients with ncHR-HPV after treatment had recurrence or metastasis, and HPV reinfection was not detected in any of them. This study suggested that HR-HPV monitoring is necessary for ppHR-HPV patients after treatment but may not be for ncHR-HPV patients.

HPV infection has been identified as a necessary but not sufficient cause of cervical cancer 15. Persistent and chronic infection with high-risk HPV types (including16, 18, 31, 33, 35, 39, 45, 51, 52, 56, 58, 59, 68, 73 and 82) were observed in almost cervical cancers 16. Over the past decades, a large number of studies focused on the relationship between HPV status and prognosis of cervical cancer 17-20. Lei J et al 17 performed a nationwide population-based study of cervical cancer cases tested for 13 HR-HPV types, and found that women with HR-HPV-positive tumors had 39% lower excess of mortality than women with HR-HPV-negative tumors. Furthermore, a meta-analysis that included 2,838 cases showed that positive HPV DNA before treatment was associated with better OS and PFS than negative HPV DNA in patients with cervical cancer 18. Besides, Barreto CL 20 reported that the HPV status had no statistically significant effect on the survival of cervical cancer patients. So, there are still controversies about HPV status and prognosis of cervical cancer.

However, few studies focus on the relationship between conversion of HR-HPV status after treatment and prognosis in patients with cervical cancers. Only four studies with good quality assessment involved HPV status before and after treatment. However, vast variation in the HPV detection rate was observed in these studies 21. Methods of HPV detection and radiotherapy may be attributed to the disparities, because three of the four studies were performed 10 years ago. Only one study by Mahantshetty et al. was conducted in the last 3 years, however HPV16/18 was defected but not all the HR-HPV and the follow-up was only 2 years 22. In our research, we found that ncHR-HPV was associated with better OS and PFS for cervical cancer. The patients with ppHR-HPV demonstrated worse outcome. We speculated that patients with persistent HPV infection may be insensitive to treatment or already have micrometastases. Besides, patients with persistent HPV infection may have increased risk of developing disease recurrence. Nagai et al. reported that persistence of HPV infection was an alarm for disease recurrence after therapeutic conization for CIN 3 23. This may be because persistent HPV infection can up-regulate the expression of HPV E6/E7 oncogene and thereby initiate carcinogenesis 24. HPV E6/E7 oncogene can degrade p53 and inactivate pRb, which maintain the growth of tumors.

Since patients whose HR-HPV status was changed to negative had a better prognosis, would that mean that we should need to take interventions for patients with persistent HP-HPV infection? HR-HPV persistence has the ability to effectively downmodulate innate immune response and can delay the development of adaptive immunity. Then, delayed immune response to HR-HPV increases the probability to transform epithelial cells. At this stage, elimination of the virus can never prevent cancer development 25-26. This indicates not only the preventive HPV vaccination, but also therapeutic HPV vaccines, which can induce cellular immune response against HPV oncoproteins and eliminate malignant cells expressing HPV proteins are urgently needed. Currently, several therapeutic vaccines, including recombinant protein vaccines, peptide vaccines, chimeric vaccines, nucleic acid vaccines are being studied for efficacy and safety 27-29. We look forward to the early clinical use of these vaccines.

In our study, we found that ppHR-HPV after treatment was associated with recurrence or metastasis, which indicated that HR-HPV monitoring is necessary for ppHR-HPV patients after treatment. However, 12 patients with ncHR-HPV after treatment had recurrence and metastasis, but for none of them HPV reinfection was detected at the time of recurrence and metastasis. Therefore, for patients with ncHR-HPV after treatment, continued HPV monitoring may not predict recurrence or metastasis. More large-scale prospective research should be carried out to explore it.

There were some limitations in this study. Firstly, this study was a retrospective study with a small sample size, so a prospective study with a larger sample size should be needed to further validate the current conclusions. Another limitation of this study was that we did not analyze the impact of different HPV genotype and multiple genotypes of HPV. Chennai et al. reported that infection with multiple genotypes was associated with poor prognosis and early recurrences in comparison to a single genotype 30. However, this study indicated that molecular detection of HR-HPV conversion status may facilitate early diagnosis of residual and early recurrent cancers after radiotherapy.

## Conclusions

In conclusion, this study demonstrated that ncHR-HPV after treatment in cervical cancer patients indicated better PFS and OS, while ppHR-HPV indicated worse prognosis and high risk of recurrence or metastasis. Therefore, we suggest that HR-HPV monitoring is necessary for ppHR-HPV patients after treatment but may not be for ncHR-HPV patients. However, further large and multi-center prospective studies should be performed to confirm these findings.

## Figures and Tables

**Table 1:** Baseline characteristics (n=112)

Variables	n	%
**Age (years)**		
>60	30	26.79
≤60	82	73.21
**Pathological type**		
Squamous carcinoma	104	92.86
Adenocarcinoma	5	4.46
Small cell carcinoma	1	0.89
Adenosquamous carcinoma	2	1.79
**FIGO Stage**		
Stage IA-IIA2	69	61.61
Stage IIB-IV	43	38.39
**Treatment**		
Surgery	91	56.8
CCRT	21	43.2

**Table 2:** Clinicopathological variables of 112 cervical cancer patients according to HR-HPV conversion status after treatment

Variables	ncHR-HPV	ppHR-HPV	*P*	*χ*^2^
**Age (years)**				
>60	22 (19.6)	6 (5.4)	0.802	0.616
≤60	62 (55.4)	22 (19.6)		
**Pathological type**				
Squamous carcinoma	78 (69.6)	26 (23.2)	1.000	0.000
Adenocarcinoma	4 (3.6)	1 (8.9)		
Small cell carcinoma	0 (0)	1 (8.9)		
Adenosquamous carcinoma	2 (1.8)	0 (0)		
FIGO Stage (Stage IA-IIA2)	52 (46.4)	18 (16.1)	0.823	0.051
Stage IIB-IV	32 (26.8)	10 (8.9)		
**Treatment**				
Surgery	69 (61.6)	23 (20.5)	1.000	0.000
CCRT	15 (13.4)	5 (4.5)		

**Table 3:** Univariate and Multivariate analysis for PFS

	Univariate analysis			Multivariate analysis		
	HR	95% CI	*P*	HR	95% CI	*P*
Age (≤60/>60)	0.94	0.39-2.28	0.89			
Pathological type (Squamous/Non-Squamous carcinoma)	0.85	0.18-3.99	0.82			
FIGO Stage(IA-IIA2/IIB-IV)	0.52	0.23-1.18	0.09			
Treatment (Surgery/CCRT)	0.29	0.10-0.90	0.002	3.57	1.57- 8.11	0.002
HPV status (nc/ppHR-HPV)	0.21	0.08-0.53	<0.001	5.14	2.32-11.46	<0.001

**Table 4:** Univariate and Multivariate analysis for OS

	Univariate analysis			Multivariate analysis		
	HR	95% CI	*P*	HR	95% CI	*P*
Age (≤60/>60)	0.53	0.18-1.54	0.19			
Pathological type (Squamous/Non-Squamous carcinoma)	1.17	0.18-7.72	0.88			
FIGO Stage (IA-IIA2/IIB-IV)	0.46	0.17-1.20	0.10			
Treatment (Surgery/CCRT)	0.35	0.10 -1.27	0.03			
HPV status (nc/ppHR-HPV)	0.08	0.03-0.26	<0.001	12.56	3.54-44.65	<0.001

**Table 5:** HR-HPV status after treatment and recurrence or metastasis

	n	ncHR-HPV	ppHR-HPV	*P*	*χ*^2^
progressive disease	25	12	13		
stable disease	87	74	13	<0.001	14.827
